# Functional Activation-Informed Structural Changes during Stroke Recovery: A Longitudinal MRI Study

**DOI:** 10.1155/2017/4345205

**Published:** 2017-10-24

**Authors:** Zhiyuan Wu, Lin Cheng, Guo-Yuan Yang, Shanbao Tong, Junfeng Sun, Fei Miao

**Affiliations:** ^1^Department of Interventional Radiology, Ruijin Hospital, Shanghai Jiao Tong University School of Medicine, Shanghai 200025, China; ^2^School of Biomedical Engineering, Shanghai Jiao Tong University, Shanghai 200030, China; ^3^Shanghai Med-X Engineering Research Center, School of Biomedical Engineering, Shanghai Jiao Tong University, Shanghai 200030, China; ^4^Department of Radiology, Ruijin Hospital, Shanghai Jiao Tong University School of Medicine, Shanghai 200025, China

## Abstract

**Objective:**

Neuroimaging studies revealed the functional reorganization or the structural changes during stroke recovery. However, previous studies did not combine the functional and structural information and the results might be affected by heterogeneous lesion. This study aimed to investigate functional activation-informed structural changes during stroke recovery.

**Methods:**

MRI data of twelve stroke patients were collected at four consecutive time points during the first 3 months after stroke onset. Functional activation during finger-tapping task was used to inform the analysis of structural changes of activated brain regions. Correlation between structural changes in motor-related activated brain regions and motor function recovery was estimated.

**Results:**

The averaged gray matter volume (aGMV) of contralesional activated brain regions and laterality index of gray matter volume (LI_GMV_) increased during stroke recovery, and LI_GMV_ was positively correlated with Fugl-Meyer Index (FMI) at initial stage after stroke. The aGMV of bilateral activated brain regions was negatively correlated with FMI during the stroke recovery.

**Conclusion:**

This study demonstrated that combining the stroke-induced functional reorganization and structural change provided new insights into the underlying innate plasticity process during stroke recovery.

**Significance:**

This study proposed a new approach to integrate functional and structural information for investigating the innate plasticity after stroke.

## 1. Introduction

Innate physiological and structural plasticity, which usually started from the several days immediately after stroke onset up to years, were reported to be the fundamental process underlying motor function recovery after stroke [1–6]. Numerous studies have looked into the process from different aspects using neuroimaging techniques. Functional magnetic resonance imaging (fMRI) data of stroke patients showed hyperactivation in contralesional motor regions and secondary motor regions which were not normally involved in motor tasks for health subjects [7–11]. The contralesional activation was also related to the severity of the motor function impairment. For example, patients with severe motor deficits exhibited higher activation in contralesional primary motor and premotor area, which was not found in the patients with mild motor deficits [12]. Longitudinal neuroimaging studies on subacute stroke patients found that the initial hyperactivation in contralesional and secondary motor regions gradually diminished and shifted to ipsilesional sensorimotor areas [3, 7, 13]. Moreover, brain reorganization also manifested in the change of connectivity between different brain regions [14, 15], such as suppressed bidirectional ipsilesional effective connectivity between supplementary motor area (SMA) and primary motor area (M1) [16] and alteration of interhemispheric functional connectivity between the bilateral motor areas [17]. Dynamic reorganization of brain network in terms of topological configuration has also been reported [18–21].

In addition to the functional plasticity, structural changes of brain after stroke have also been investigated. After acute ischemic stroke, delayed brain atrophy, accompanied by a motor function improvement, was found in several brain areas structurally connected with the region of lesions [22]. Cortical thickness was reported to reduce in ipsilesional M1 for stroke patients [23]. According to voxel-based morphometry (VBM), decrease of gray matter volume (GMV) in intact motor regions was correlated with the motor deficit during recovery [24]. In addition, longitudinal studies have investigated the structural plastic change after stroke and evaluated its correlation with the motor function recovery [25–27]. For example, Fan and colleagues reported decrease of the bilateral GMV around lesions and increase of the GMV in hippocampus and precuneus, which were positively correlated with motor function recovery, through 5 recording time points from acute stage (<5 days after stroke onset) to chronic stage (1 year later) [25]. In addition, Dang and colleagues found GMV decreased in ipsilesional SMA but increased in contralesional SMA, and the changes of GMV were correlated with the motor function recovery during subacute stage [26]. Moreover, structural reorganization in contralesional cognitive-related cortices was suggested to contribute to motor recovery after subcortical stroke based on data recorded in acute stage and chronic stage [27].

Though progress has been made in studying the innate physiological and structural plasticity, the correlation between functional reorganization and structural change is still not clear. Human brain is a large-scale network in both functional and structural domain. Both functional and structural change are important to understand the brain reorganization during stroke recovery [28, 29]. Schaechter and colleagues suggested that structural plasticity was colocalized to regions with functional plasticity after stroke [30]. Combining the functional reorganization and the structural changes after stroke would provide new insights into the innate physiological and structural plasticity. However, few longitudinal studies have been reported from this perspective so far to the best of our knowledge. And technically, previous studies usually adopted general linear model (GLM) to identify voxels with GMV changes in the whole brain. Such a method could be subjected to type I error and should be solved by multiple comparison correction [31]. Furthermore, this method did not take the heterogeneity of lesion (e.g., location-dependent injury severity) into account as the group-level GLM analysis assumes the consistency of stroke-induced GMV changes across patients with heterogeneous lesions. In this study, thus we focused on the changes of averaged GMV in the activated brain regions instead of the whole brain, so as to control the type I error, and achieved an individualized GMV study regarding the different lesion locations in stroke patients.

With the above consideration, this study aimed to combine the functional reorganization and structural change and investigate the dynamic changes of GMV in the activated brain regions and their correlation with motor function restoration after stroke. In addition, previous studies reported that spontaneous recovery tended to occur within the first 3 months after stroke onset, which is considered as the critical period for stroke rehabilitation [1, 32, 33]. During the critical period, brain was reorganized dramatically from both functional and structural perspectives [1, 34]. Therefore, twelve stroke patients were recruited to have MRI scanning at four consecutive time points during the first 3 months after stroke onset. Functional activation during finger-tapping task was used to inform the analysis of structural changes. In addition, the correlation between structural changes of activated brain regions and motor function recovery was also analyzed.

## 2. Materials and Methods

### 2.1. Subjects

Twelve stroke patients (male/female: 9/3; age: 61.5 ± 8.8 years), recruited from the Rui Jin Hospital, Shanghai Jiao Tong University School of Medicine, participated in the study at four time points after stroke (i.e., less than 10 days (P1, 5.1 ± 2.4 days), two weeks (P2, 13.9 ± 0.5 days), one month (P3, 30.5 ± 2.3 days), and three months (P4, 90 days)), respectively. Starting from P2, some patients quit the fMRI scans in the follow-ups; that is, two of twelve patients quit at P2, five patients quit at P3, and seven patients quit at P4, which were listed in Table 1. The demographic and clinical data were listed in Table 1. Inclusion criteria of stroke patients were (i) age from 45 to 80 years, (ii) right handedness, (iii) first-onset ischemic stroke with motor deficits, (iv) no history of neuropsychiatric diseases, epilepsy, cerebral vascular abnormalities, and trauma, and (v) Fugl-Meyer Index (FMI, a clinical measure, which indicates motor function of upper and lower limbs, ranging from 0 to 100. Higher FMI score indicates better motor function) [35] at P1 ranging from 50 to 95. Fifteen age-matched and gender-matched right handed healthy subjects (male/female: 7/8; age: 63 ± 7.2) were recruited as the control group. The Ethics Committee of Rui Jin Hospital approved the experiment protocols, and every participant gave an informed consent before experiment. Note that the results of functional brain networks with this dataset have been reported in another paper [19].

### 2.2. Task Design

The patients took two consecutive block-design finger-tapping sessions with a 15 min interval at each of the four time points (i.e., P1, P2, P3, and P4) after stroke, respectively. Patients performed the finger-tapping task (i.e., tapping the thumb with other fingers one time per second) with their unaffected hands (the hand ipsilateral to the hemisphere with lesion) in the first session and then with their affected hands (the hand contralateral to the hemisphere with lesion) in the second session. Subjects in control group performed the same experiment only once, with the left hand task in the first session followed by the right hand task in the second session with a 15 min interval. Each session consisted of a resting block (30 s) alternated with a task block (30 s) for three repetitions, preceded by 12 s of preparing period. During the resting blocks, subjects laid still and remained motionless, relaxing, and awake; while under motor-task block, subjects performed the finger-tapping task. Before the task, T1-weighted (T1W) and T2-weighted (T2W) were acquired for each subject.

### 2.3. Data Acquisition

**Figure 1:**
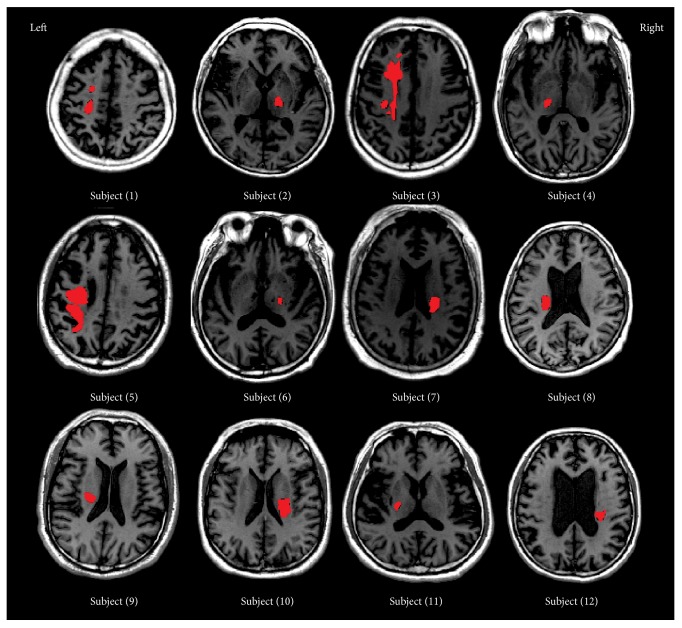
Lesions of 12 stroke patients, manually outlined using MRIcron software by a neurologist.

All images were acquired with a 1.5 Tesla MRI scanner (Excite HD, General Electric Medical System, Milwaukee, WI, USA) and an 8-channel NVHEAD coil in Rui Jin Hospital. The head of the participant was snugly fixed by a foam pad to reduce head movements and scanner noises. Structural MRI data were acquired using a T1W fast-spin-echo (FSE) sequence: 32 axial slices, thickness/gap = 5/0 mm, repetition time (TR) = 2180.0 ms, echo time (TE) = 28.7 ms, field of view (FOV) = 240 mm × 180 mm, matrix = 320 × 224, and the scanning time was 3 min 10 s. T2W images were acquired using a FSE sequence: 32 axial slices, thickness/gap = 5/0 mm, TR = 4350 ms, TE = 102 ms, FOV = 240 mm × 180 mm, matrix = 384 × 256, and the scanning time was 3 min 37 s. During the block-design sessions, fMRI BOLD data were acquired from the top of the brain to the lower part of the medulla oblongata, using an echo-planar imaging (EPI) sequence: 32 axial slices, thickness/gap = 5/0 mm, TR = 3000 ms, TE = 60 ms, flip angle = 90°, FOV = 240 mm × 180 mm, matrix = 64 × 64, and the scanning time was 3 min 12 s. For stroke patients, lesions were manually outlined using MRIcron software (http://people.cas.sc.edu/rorden/mricron/) at P1 by a neurologist (Figure 1).

### 2.4. VBM Analysis

The T1W structural data were analyzed using the voxel-based morphometry (VBM) technique in SPM8 (Wellcome Trust Centre for Neuroimaging, University College London, London, UK) with MATLAB [36] following previous studies [36, 37]. In order to avoid the adverse impact of lesions on data processing, lesions were masked out in the following data processing. VBM analysis included two main steps. First, a customized GM template was created from healthy control group based on their GM probability map following the methods in previous studies [25, 26], including segmentation, normalization to Montreal Neurological Institute (MNI) space, averaging, and smoothing by a Gaussian filter with a full-width at half-maximum (FWHM) smoothing kernel of 8 mm. In the second step, the follow-up T1W images of each subject were coregistered to their corresponding T1W images obtained during the first recording using the standard SPM registration method, which started with an affine linear registration and then proceeded with a nonlinear registration using a warping transformation model consisting of a linear combination of low-frequency periodic basis functions [38]. Then the GM images of patients were obtained from segmentation and were further nonlinear-normalized into the MNI space using the customized template obtained in the first step by the Diffeomorphic Anatomical Registration Through Exponentiated Lie Algebra (DARTEL) algorithm [39]. Since the patients' GM images might be distorted and influenced by the lesions, the DARTEL algorithm was adopted in the second step to increase the accuracy of coregistration, which has been applied in studies of pathological images [26, 40]. After that, normalized GM images were modulated using the Jacobian determinant to preserve the total amount of GM and then were smoothed using a Gaussian filter with FWHM of 8 mm. In addition, global normalization was performed to adjust the GMV according to the corresponding intracranial volume. After these steps, a GMV map, which denotes the GMV for each voxel, was generated for each subject and each time point.

### 2.5. Identification of Activated Brain Regions during Motor Task

**Figure 2:**
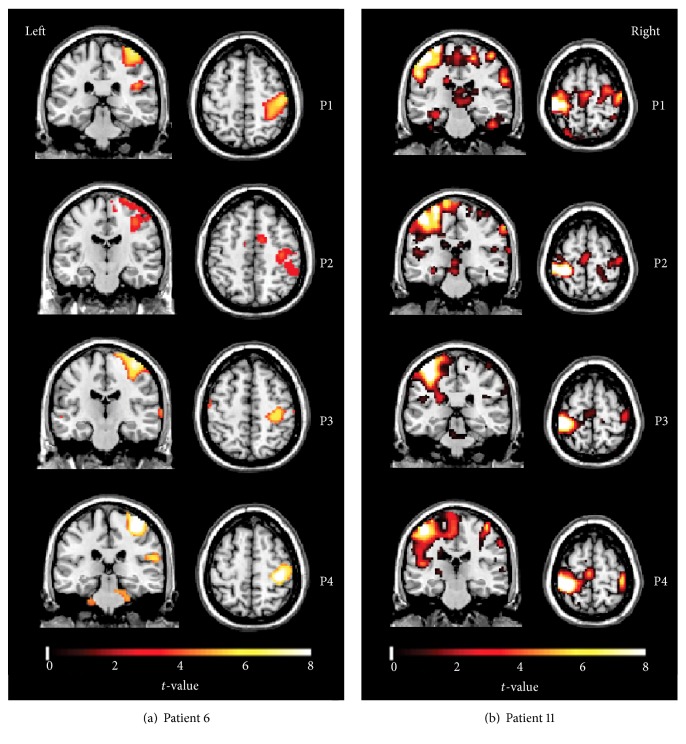
Activation of patient number 6 (a) and patient number 11 (b) while they were performing the finger-tapping task with the affected hands (left hand for patient number 6; right hand for patient number 11) at four time points after stroke (*p* < 0.001, extent threshold = 13 voxels). Colorbar represents the* t*-value. Both patients had lesions in basal ganglia.

Before identifying the activated brain regions during motor task, the fMRI BOLD was preprocessed. For each subject, the data of the first 12 s preparing period (4 volumes) in each session were discarded to avoid the magnetization equilibrium effects and allow the subjects to adapt to the experiments inside the scanner. The remaining fMRI data (60 volumes) were preprocessed with SPM8 (Wellcome Trust Centre for Neuroimaging, University College London, London, UK) including spatial realignment to the mean volume of a series of images, coregistration, spatial normalization to the Montreal Neurological Institute (MNI) template using the unified segmentation approach [41], and spatial smoothing (8 mm isotropic kernel). Then, the preprocessed fMRI BOLD data were statistically analyzed using GLM in SPM8. For every subject at each session, the box-car vectors for task state were convolved with a hemodynamic response function and included into the design matrix. In addition, the head movement parameters were used as covariates to remove the variance induced by head motions, and the default temporal frequency cut-off (128 sec high pass) in SPM8 was used. After that, the GLM was used to obtain the individual activation maps (*p* < 0.001, extent threshold = 13, which was determined by the Monte Carlo simulations with the program AlphaSim in AFNI [42, 43]) for each subject. Since our main aim was to examine the stroke-induced changes, we focused on the activated brain regions when performing task with the affected hands, no matter whether it was right hand (7 patients had right hand affected) or left hand (5 patients had left hand affected).  Figure 2 illustrated the activation of patient 6 (mild motor impairment) and patient 11 (severe motor impairment) at four time points after stroke. We also performed additional investigation on the activation when moving the nonaffected hand and found that the activation mainly located in the nonaffected motor cortex (please see Fig. S1 in Supplementary Material available online at https://doi.org/10.1155/2017/4345205), which was similar to those of the healthy controls. The results are also in line with findings of previous studies [12, 44]. For healthy controls, the activated brain regions were analyzed during both right hand session and left hand session and were further used in the comparison with patients.

### 2.6. GMV of Activated Brain Regions

In order to quantify the dynamic changes of GMV in activated brain regions during stroke recovery, mask of activated brain regions was obtained for each subject at each time point and then was used to extract the values of voxels in the activated brain regions from the corresponding GMV image obtained from VBM analysis. The mean of those values was considered as the averaged GMV in the activated brain regions. Actually, we did not calculate the averaged GMV for a single activated brain region but calculated the averaged GMV for activated brain regions in ipsilesional hemisphere, contralesional hemisphere, or bilateral hemispheres instead. Note that we did not refer to averaged GM density of a single lesion as we masked out the lesion in the data processing and analysis. We used individual-level activated brain regions to extract the GMV, so that the heterogeneous lesion locations of patients could be reflected in their individual-level activated brain regions and thereby were taken into account in the following GMV analysis. Note that previous studies also performed fMRI data analysis, such as activation laterality analysis, based on individual-level activated brain regions [45, 46]. Numerous studies indicated that stroke would induce both functional and structural reorganization of the whole brain [18, 25]. So we would like to examine the changes of averaged GMV of bilateral activated brain regions (aGMV) through stroke recovery. In addition, studies also found that the reorganizations of two hemispheres were distinct [7, 12], which made us look into the changes of averaged GMV of contralesional and ipsilesional activated brain regions (aGMV_*c*_ and aGMV_*i*_). The aGMV was calculated for each subject at each time point as follows:

(1) $$\begin{array}{c} aGMV=\frac{\sum_{i=1}^{N}v_{i}}{N}, \end{array}$$

where *N* represents the number of voxels in activated brain regions and *v*_*i*_ represents the value of the *i*th voxel in the corresponding GMV image obtained from VBM analysis. In addition, the dynamic changes of activated brain regions volumes were also analyzed. Please note that the number of voxels in the activated brain regions (*N*) does not represent the volume of an activated brain region but specifically the volume of an activated brain region warped to the MNI space. We regarded the *N* as the indication of the volume of activated brain regions under two considerations: (i) This study is a longitudinal study, targeted to estimate the trends of several measurements over time. Since the head sizes of patients would not change dramatically for three months, it is reasonable to say that the changes in *N* during the recovery time were due to changes of activation volumes and thus can indicate the changes of activation volumes in the subjects' native space; (ii) using *N* of normalized activated brain regions can also take the intersubject variability of head sizes into account. In addition, several previous studies, which estimated the changes of activation volume, also used the number of voxels in the normalized images to represent the volume of activated brain regions [47–49]. Thus, *N* refers to the volume of activation regions for simplification hereafter.

In addition, the interhemispheric balance of GMV in bilateral activated brain regions was used to assess how brain would reorganize structurally during stroke recovery. Thus, laterality index of GMV for activated brain regions was calculated according to the following equation:

(2) $$\begin{array}{c} {LI}_{GMV}=\frac{\left({aGMV}_{c}-{aGMV}_{i}\right)}{\left({aGMV}_{c}+{aGMV}_{i}\right)}, \end{array}$$

where aGMV_*c*_ and aGMV_*i*_ represent the averaged GMV of contralesional activated brain regions and ipsilesional activated brain regions, respectively. LI_GMV_ ranges from +1 to −1 with positive LI_GMV_ indicating higher averaged GMV in contralesional activated brain regions and negative LI_GMV_ representing higher averaged GMV in ipsilesional activated brain regions. In order to be consistent across patients, for healthy subjects, aGMV_*c*_ represents the averaged GMV of activated brain regions ipsilateral to the moving hand (i.e., contralesional side for patients is ipsilateral to their affected hands), and aGMV_*i*_ represents the averaged GMV of activated brain regions contralateral to the moving hand (i.e., ipsilesional side for patients is contralateral to their affected hands).

For the volume of activated brain regions, the laterality index was calculated as follows:

(3) $$\begin{array}{c} {LI}_{N}=\frac{\left(N_{c}-N_{i}\right)}{\left(N_{c}+N_{i}\right)}, \end{array}$$

where *N*_*c*_ and *N*_*i*_ represent the number of voxels in contralesional activated brain regions and ipsilesional activated brain regions, respectively.

### 2.7. Statistical Analysis

Linear mixed-effects models were employed to quantify the dynamic changes of GMV (i.e., aGMV, aGMV_*c*_, and aGMV_*i*_) and LI_GMV_, as well as their correlation with FMI. Linear mixed-effects model takes advantages of all available data, including those from the patients who missed some follow-ups [50]. In analyzing the correlation of GMV and LI_GMV_ with the days after stroke, all patients were assumed to have a common slope and a fixed effect term. In addition, a random effect term was introduced to allow for variations across different patients. The ages of patients were also included in the model as a covariate to regress out its effect. The model is as follows:

(4) Gij=μ+bi+Tijβ1+Aiβ2+Tij:Aiβ3+εij,i=1,2,3,…,K;  j=1,2,3,4,

where *G*_*ij*_ represents either the GMV or LI_GMV_ of the *i*th subject from the* j*th scan (up to four scans in this study), *μ* is the fixed effect term for all patients, *b*_*i*_ is the random effect term for each patients, *T*_*ij*_ represents the days after stroke and *β*_1_ is its scalar (the common slope), *A*_*i*_ are the ages of patients and *β*_2_ is its scalar, (*T*_*ij*_ : *A*_*i*_) represents the interaction between these two factors and *β*_3_ is the scalar of the interaction term, *ε*_*ij*_ represents the residual error of the model, and *K* is the number of patients.

Furthermore, previous studies reported that lesion volume diminished significantly during stroke recovery [51–53]. In order to test whether our data would result in the similar trend of diminishing lesion volume, the dynamic changes of lesion volumes was also examined using (4).

For correlation of aGMV and LI_GMV_ with FMI, except for ages, the days after stroke were also included in the model as a covariate. Since the days after stroke at scans were different across patients, the effect of days after stroke should be taken into account. The model is as follows:

(5) Fij=μ+bi+Gijβ1+Aiβ2+Tijβ3+Tij:Aiβ4+εij,i=1,2,3,…,K;  j=1,2,3,4,

where *F*_*ij*_ represents the FMI, *G*_*ij*_ represents either aGMV or LI_GMV_ of the *i*th subject from the* j*th scan, and other terms represent the same variables as in (4).

Note that identical analysis was performed for *N*, *N*_*c*_, *N*_*i*_, and LI_*N*_ to examine their correlation with days after stroke (by (4)) or FMI (by (5)). Furthermore, in order to analyze the relationship of GMV as well as LI_GMV_ with activated regions volume, a linear mixed-effects model was employed as follows:

(6) Gij=μ+bi+Nijβ1+εij,i=1,2,3,…,K;  j=1,2,3,4,

where *G*_*ij*_ represents either the GMV or LI_GMV_ of the *i*th subject from the* j*th scan, *N*_*ij*_ represents the activated regions volume (i.e.,* N*, *N*_*c*_, and *N*_*i*_) of the *i*th subject from the* j*th scan, and other terms represent the same variables as in (4) and (5).

In addition, to quantify the correlation of aGMV/LI_GMV_ with FMI at the initial stage P1, a linear regression model was employed and optimized by backward-stepwise model selection [54]. The optimal model includes the ages of patients as a covariate as follows:

(7) Fi=μ+Giβ1+Aiβ2+Gi:Aiβ3+εi,i=1,2,3,…,K,

where *F*_*i*_ represents the FMI of the *i*th patient at P1, *G*_*i*_ represents either the aGMV or LI_GMV_ at P1 for the *i*th patient, and *A*_*i*_ is the age of the *i*th patient.

## 3. Results

### 3.1. Dynamic Changes of aGMV_*c*_ and LI_GMV_ after Stroke

**Figure 3:**
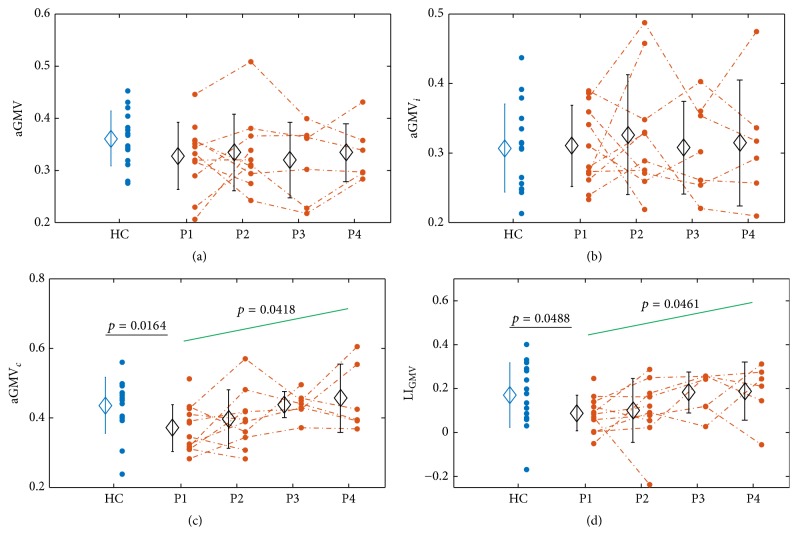
Illustration of aGMV (a), aGMV_*i*_ (b), aGMV_*c*_ (c), and LI_GMV_ (d) of healthy controls (HC) and patients at four time points (i.e., P1, P2, P3, and P4) after stroke. Each dot represents one subject at the corresponding time point, the dot line links the dots belonging to the same patient, and error bars indicate the standard deviations across HC and stroke patients at four time points, respectively. Green lines indicate the change trend across time points.

First of all, the dynamic changes of FMI from P1 to P4 were examined (please see Supplementary Materials for details), and significant increase of FMI was identified (*p* < 0.0001). After that, dynamic changes of aGMV, aGMV_*i*_, aGMV_*c*_, and LI_GMV_ after stroke were examined using the model in (4). Note that the subject-wise values for aGMV, aGMV_*i*_, aGMV_*c*_, LI_gmv_, *N*, *N*_*i*_, *N*_*c*_, and LI_*N*_ were provided in the Supplementary Materials (Table S1). In addition, the comparisons between healthy controls and patients were also performed using two-sample* t*-test. The results are illustrated in Figure 3 and Table 2. aGMV_*c*_ and LI_GMV_ of patients were significantly smaller than healthy controls at P1 (aGMV_*c*_: *p* = 0.0164; LI_GMV_: *p* = 0.0488) and then gradually increased from P1 to P4 (aGMV_*c*_: *p* = 0.0418; LI_GMV_: *p* = 0.0461). Note that the results illustrated in Figure 3 were based on the activated brain regions of healthy controls when they were performing tasks using right hands. An additional analysis was conducted based on the activated brain regions of healthy controls when they were performing tasks using left hands, and the results of the comparisons between patients and healthy controls were similar to those illustrated in Figure 3. aGMV_*c*_ and LI_GMV_ of patients were still significantly smaller than those of healthy controls at P1 (aGMV_*c*_: *p* = 0.0105; LI_GMV_: *p* = 0.0358). However, for aGMV or aGMV_*i*_, no significant differences were found between patients and healthy controls, and no significant dynamic changes were found from P1 to P4 either.

For activated brain regions volume, similar analyses were performed for *N*, *N*_*c*_, *N*_*i*_, and *LI*_*N*_, and no significant results were found. In addition, the relationship of aGMV, aGMV_*i*_, aGMV_*c*_, and LI_GMV_ with activated brain regions volume was examined using the model in (6). Statistical results are illustrated in Table 3. LI_GMV_ was negatively correlated with *N*_*c*_ (*p* = 0.0094). For aGMV, aGMV_*i*_, and aGMV_*c*_, no significant correlations were identified. Though the correlation between aGMV_*c*_ and *N*_*c*_ was not significant, relatively strong negative correlation was still identified (*p* = 0.1067). These results demonstrated that the decrease in contralesional activation volume might play a critical role in the increase of aGMV_*c*_ and LI_GMV_ after stroke.

### 3.2. Correlation between aGMV and Motor Function

The linear mixed-effects model (see (5)) was employed to estimate the correlation between FMI and aGMV as well as LI_GMV_. Results showed that the FMI was negatively correlated with aGMV (*p* = 0.0319) (Table 4), which suggested that smaller aGMV is correlated to better motor function during stroke recovery. No significant correlation was found between FMI and LI_GMV_. For activated brain regions volume, no significant correlations were found.

In addition, by the linear regression model (see (7)), positive correlations were found between LI_GMV_ and FMI at P1 (*p* = 0.0028), indicating that LI_GMV_ was significantly related to the motor function after stroke at the early stage after stroke.

### 3.3. Dynamic Changes of Minimum GMV in Contralesional Activated Voxels

**Figure 4:**
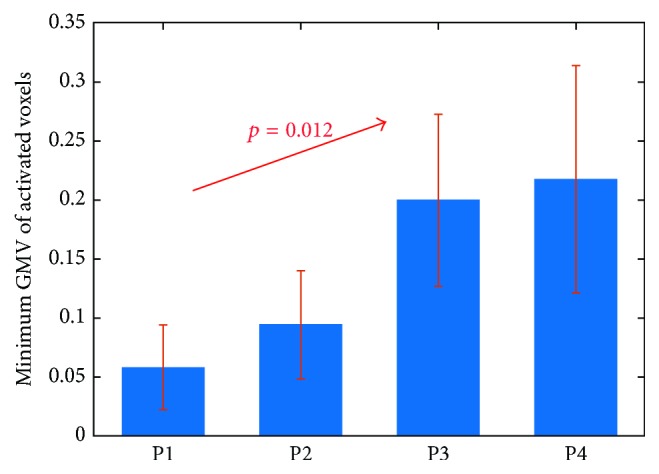
Mean minimum GMV in contralesional activated voxels across patients at P1, P2, P3, and P4, respectively. Positive correlation between minimum GMV and days after stroke was found by linear mixed model. Error bars indicate the standard deviations.

The above results demonstrated that the decrease in contralesional activation volume might play a critical role in the increase of aGMV_*c*_ and LI_GMV_ after stroke. To investigate whether the increasing aGMV_*c*_ was due to the shrinking of contralesional activation back to the brain regions with greater GMV, we examined the dynamic changes of minimum GMV in contralesional activated voxels from P1 to P4. For each subject at each time point, the minimum GMV in contralesional activated voxels was defined as the minimum GMV among activated voxels in the contralesional hemisphere. Linear mixed-effects model showed the positive correlation between the minimum GMV and days after stroke (*p* = 0.012, Figure 4), suggesting that the contralesional activation was shifting to the brain regions with greater GMV.

### 3.4. Dynamic Change of Lesion Volume after Stroke

In addition, (4) was also used to examine the dynamic change of lesion volume after stroke. Statistical analysis showed that the lesion was diminishing after stroke as the lesion volume negatively correlated with days (*p* = 0.0216, listed in Table 5).

## 4. Discussion

### 4.1. Increasing aGMV_*c*_ and LI_GMV_ during Stroke Recovery

In this study, dynamic changes of averaged GMV in activated brain regions and LI_GMV_, which indicates the interhemispheric balance of GMV in bilateral activated brain regions, were examined after stroke. LI_GMV_ of patients at P1 was weaker than that of healthy controls (Figure 3), but LI_GMV_ of patients was gradually increasing after P1 (Table 2) and eventually attained the same level as the healthy controls (Figure 3(d)). The increasing LI_GMV_ after stroke was mainly due to the increasing aGMV_*c*_, which represents the averaged GMV in contralesional activation, during the recovery (Figure 3(c)). In addition, LI_GMV_ and aGMV_*c*_ were negatively correlated with *N*_*c*_ (Table 3). Thus, in our opinion, the increasing aGMV_*c*_ might be due to the diminishing stroke-induced contralesional abnormal activation, which have relatively low GMV, during stroke recovery (Figure 4).

Previous studies suggested that ipsilateral activation depended on the communication with the contralateral hemisphere via corpus callosum, as activation diminished and even disappeared in the ipsilateral hemisphere, while contralateral activation did not change for callosotomized subjects [55–57]. In addition, though with debates, previous studies primarily supported that the corpus callosum served a predominantly excitatory component connecting homologous cortical areas in two hemispheres [58]. Thus, ipsilateral activation might be induced by the influence from the contralateral hemisphere and the ipsilateral activation mainly located in the regions connecting to corpus callosum. Specifically, the ipsilateral activated brain regions might have dense neural connections with corpus callosum and thus have high GMV, which was supported by a connectivity study [59]. As a result, for healthy subjects, normally activated ipsilateral brain regions usually possessed dense neural connections and thus high GMV, while contralateral activation covered larger brain regions, some of which had less dense neural connections than activated ipsilateral brain regions and thus resulted in lower GMV. Therefore, aGMV_*c*_ was greater than aGMV_*i*_ for healthy subjects and resulted in positive LI_GMV_, which was in line with our finding (Figure 3(d)). Here note that, for healthy subjects, aGMD_*c*_ represents the averaged GMV of activated brain regions ipsilateral to the moving hand, which is corresponding to contralesional side for patients.

However, for patients at stroke onset, contralesional activation (i.e., ipsilateral to the affected hand) usually recruited brain regions which were not normally involved in motor tasks [7–9]. In addition, these abnormally activated brain regions were different from the ipsilateral activated brain regions connected with corpus callosum as in healthy controls. Instead, they might be related to the contralesional neural pathways which were activated to compensate the impaired ipsilesional neural pathways [60, 61]. Therefore, the abnormally activated brain regions did not possess dense neural connections as the ipsilateral activated brain regions in healthy controls. As a result, aGMV_*c*_ decreased at stroke onset, resulting in smaller LI_GMV_ than that of healthy controls. However, during stroke recovery, the contralesional abnormal activation diminished and gradually shifted back to the brain regions with dense neural connections with corpus callosum like healthy controls. This process was indicated by the increasing minimum GMV in contralesional activated voxels through P1 to P4 (Figure 4), because the increasing minimum GMV suggested that the contralesional activation shifted to regions with higher GMV. As a result, aGMV_*c*_ increased during the recovery, thus yielding the increasing of LI_GMV_.

Furthermore, our results also suggested that LI_GMV_ at P1 was positively correlated with motor function at the stroke onset, as indicated by FMI at P1. Patients with severe motor impairment usually showed more extended abnormal activation in contralesional hemisphere [7–9], covering brain regions with relatively lower GMV, thus resulting in lower aGMV_*c*_ and LI_GMV_, which was supported by the negative correlation between LI_GMV_ and *N*_*c*_ (Table 3). However, patients with mild motor impairment usually had similar contralesional activation to healthy controls [12], which was mainly located in the regions with dense neural connections and thus higher GMV, as we discussed in Section 4.1. As a result, the patients with mild motor impairment had higher aGMV_*c*_ and thus higher LI_GMV_. The results were also supported by the findings that healthy controls have higher aGMV_*c*_ and LI_GMV_ than patients (Figure 3).

### 4.2. Negative Correlation between aGMV and FMI

When analyzing the correlation between structural changes in activated brain regions and motor function recovery, aGMV, which represents the averaged GMV in bilateral activation, was negatively correlated with FMI during the stroke recovery (Table 4). In our opinion, such a negative correlation between aGMV and FMI was likely due to the extended activation in contralesional hemisphere. Studies had suggested that GMV in ipsilesional motor-related brain regions decreased after stroke [23–26], while the GMV in some contralesional brain regions, such as SMA, was even reported to increase [26]. Therefore, we speculated that the GMV of contralesional motor-related brain regions was higher than that of ipsilesional motor-related brain regions after stroke, as shown by the positive LI_GMV_ during stroke recovery (Figure 3(d)). From the functional aspect, patients with severe motor impairments usually showed more extended contralesional activation than those with mild motor impairments [34, 62], as demonstrated in Figure 2. Because of the higher contralesional GMV, more extended contralesional activation would increase the weight of contralesional GMV in aGMV, which leads to higher aGMV. Therefore, with the stroke recovery, patients with lower FMI (more severe motor impairment) would usually have more extended contralesional activation and thus would have greater aGMV, resulting in a negative correlation between FMI and aGMV. The results of aGMV, estimated based on both functional and structural information, further suggested that combining the functional and structural reorganization would provide new insights into the underlying innate plasticity process during stroke recovery.

With respect to the lesion volume, previous studies reported that lesion volume diminished significantly during stroke recovery [51–53]. In this study, the dynamic change of lesion volume during stroke recovery was also examined and the lesion was diminishing after stroke as the lesion volume negatively correlated with days after stroke (Table 5), which was in line with previous studies regarding lesion volume changes [51–53] and further demonstrated reliability of findings in this study in an indirect way.

In this study, we used VBM to analyze the structural changes during stroke recovery. Tensor-based morphometry (TBM) approach could outperform the VBM in some situations. However, according to previous studies, TBM, which is a high degree of freedom method, relies on a high degree of registration accuracy and may be underpowered in situations where registration accuracy is lower [63, 64]. Our study is a disease-specific analysis, which results in very different spatial atrophy profiles due to lesions, and, thus, increases the uncertainty in registration. A VBM approach would be suitable in the case with more uncertainty in registration [64]. Therefore, we used a VBM approach rather than a TBM approach in this study.

To the best of our knowledge, this is the first study that combines the stroke-induced functional reorganization and the structural changes to examine the longitudinal changes of brain plasticity during stroke recovery. Compared with longitudinal functional reorganization studies [7–9], this study took the structural changes into account and would be more effective in revealing the relationship between stroke recovery and brain reorganization. In contrast to structural changes through voxel-wise GLM analysis of the whole brain [25, 26], this study focused on the structural changes of the functional activation brain regions. In addition, this study mainly focused on the dynamic changes of averaged GMV in activated brain regions, which were not subjected to the type I error induced by multiple comparison like in voxel-vise analysis. Furthermore, the activation map was created for each subject and the individualized activation maps were able to reflect intersubject variability of lesion locations and impairment severities. As a result, the structural changes based on the individualized activation maps took the heterogeneity of lesion in stroke patients into account. Therefore, for the first time, this study integrated functional and structural information and demonstrated that this method did provide new insights into the innate physiological and structural plasticity during stroke recovery and overcome the heterogeneous lesion locations. However, due to the difficulty of collecting longitudinal data of stroke patients, the number of stroke patients in this study was relatively small, just as several previous papers which also conducted longitudinal studies on stroke recovery with sample sizes around ten [12, 18, 25, 65]. More stroke patients should be recruited to improve the statistical power and further consolidate our findings. In addition, several patients dropped out the fMRI scans in follow-ups. So we employed the linear mixed-effects regression model which is quite robust to missing data and can take advantages of all available data from each subject [50]. Nevertheless, if we could have the complete data from all follow-ups, we could do more statistical analysis such as paired* t*-test and ANOVA to further validate our findings. Since the 7 patients dropped out in P4, we did an additional analysis for the data of the first three time points using the identical analysis method used for the data of all four time points and found similar results to those of all four time points (please see Supplementary Materials for details). In addition, since our data only covered 3 months after stroke onset, which was still subacute phase, we were not able to explore the stroke recovery from the long-term perspective with these data, though such exploration would be very important for deep understanding of stroke recovery and need further study with data of longer time span in the future. The relation between lesion locations and clinical scores is another interesting topic. However, due to the limited sample size of each lesion location, we were not able to perform reliable statistical analysis based on our dataset. We expect that future study with larger dataset could address this issue reliably.

Another issue was that the patients group was a mix of patients with lesions in left hemisphere and patients with lesion in right hemisphere. In this study, the brain activation of patients was obtained based on block-design finger-tapping task performed by affected hands, no matter whether it was left hand or right hand. Previous studies reported relatively larger activation during dominant hand movement compared with nondominant hand movement [66, 67]. Thus, the inconsistent activation between patients using right hands and patients using left hands might affect the results. However, this study mainly focused on the dynamic changes of averaged GMV in activated brain regions, which were created for each subject, rather than being obtained from group-level statistical analysis. By this way, the individualized activation maps were able to reflect the intersubject variabilities of lesion locations, impairment severities, and hand dominance. As a result, we believe that the GMV changes of the individualized activated brain regions indirectly took the different dominant hands into account. In addition, though the inconsistent activation was reported between dominant hand movement and nondominant hand movement, a lot of stroke studies, especially the longitudinal stroke studies, did not take the hand dominance into consideration [7, 12, 13, 44, 68–70]. In our opinion, the primary reason was the difficulty of recruiting stroke patients with lesions on the same hemisphere, especially for longitudinal studies with several follow-up examinations. Though the stroke patients could be divided into two groups according to the sides of lesions, the limited sample size would also make it statistically infeasible. Another reason might be that the influence of hand dominance on activation would be far less significant compared with the stroke-induced reorganization of activation. Nevertheless, if more stroke patients with lesions on the same hemisphere could be recruited in the future study, more reliable results could be obtained. In addition, with respect to the effect of hand dominance on activation and laterality index of patients, the cortical activation of stroke patients during hand movement was mainly dependent on the impairment of brain induced by stroke [7, 12, 13], and the cortical activation differences induced by hand dominance were far less significant than those induced by stroke. Thus, it would be difficult to single out the effect of hand dominance under the strong influence of stroke-induced impairment, and the intersubject variability of impairment would make it even more difficult. What is more, the limited sample sizes of patients using dominant hand (7 patients) and nondominant hand (5 patients) would make the statistical analysis unreliable. Nevertheless, if more stroke patients with similar impairment could be recruited in the future study, it would be possible to single out the effect of hand dominance on the cortical activation and laterality index for stroke patients.

## 5. Conclusions

In this study, we investigated the dynamic changes of GMV in activated brain regions and its correlation with motor function restoration after stroke with fMRI BOLD, T1W, and FMI data collected from 12 stroke patients at four consecutive time points during the first 3 months after stroke onset. With fMRI BOLD data, functional activation during finger-tapping task was obtained to inform the analysis of structural changes of activated brain regions and their relationship with motor functional recovery. Results showed that aGMV_*c*_ and LI_GMV_ increased during stroke recovery. In addition, aGMV was negatively correlated with FMI during the stroke recovery, and LI_GMV_ was positively correlated with FMI at initial stage after stroke. This study proposed a new approach to investigate the innate plasticity after stroke by integrating functional and structural information together and provided new insights into the underlying innate plasticity process during stroke recovery.

## Supplementary Material

Table S1: Subject-wise values for *aGMV*, *aGMV_i_* and *aGMV_c_*, *LI_gmv_*, *N*, *N_i_*, *N_c_*, and *LI_N_*.
Figure S1: The group-level activation map of healthy controls moving their right hands (A); The group-level activation map of stroke patients moving unaffected hands at P1 (B), P2 (C), P3 (D), and P4 (E). The threshold for activation was p < 0.001, cluster size > 13 voxels.
Table S2: Statistical Results of Linear Mixed-Effects Model for dynamic change of FMI over time.

## Figures and Tables

**Table 1:** Patient information and the Fugl-Meyer indexes at four fMRI scan time points after stroke.

Subject number	Age (yr)	Gender	Hemisphere of infarct	Location of infarct	FMI			
					<10 d	2 wk	1 mon	3 mon
(1)	77	F	Left	Parietal lobe	54 (√)	54 (√)	54	59
(2)	67	F	Right	Basal ganglia	73 (√)	77 (√)	79	96
(3)	56	M	Left	Corona radiate	71 (√)	75 (√)	75	75
(4)	69	M	Left	Basal ganglia	59 (√)	55	62	63
(5)	68	M	Left	Parietal lobe	51 (√)	50	53 (√)	57 (√)
(6)	58	M	Right	Basal ganglia	94 (√)	94 (√)	99 (√)	99 (√)
(7)	60	M	Right	Basal ganglia	63 (√)	62 (√)	71 (√)	81 (√)
(8)	60	F	Left	Body of lateral ventricle	72 (√)	78 (√)	77 (√)	86 (√)
(9)	50	M	Left	Basal ganglia	95 (√)	95 (√)	99 (√)	99
(10)	47	M	Right	Body of lateral ventricle	85 (√)	87 (√)	96	97
(11)	56	M	Left	Basal ganglia	53 (√)	55 (√)	61 (√)	66 (√)
(12)	70	M	Right	Body of lateral ventricle	93 (√)	92 (√)	99 (√)	99 (√)

M = male; F = female; yr = year; d = day; wk = week; mon = month; FMI = Fugl-Meyer Index; (√) indicates that the fMRI data of the subject were collected as well in the corresponding time point, and subjects without (√) quit the fMRI scan in the corresponding time point.

**Table 2:** Statistical results of linear mixed-effects model for aGMV, aGMV_*i*_, aGMV_*c*_, and LI_GMV_ by (4).

	Value	Std. error	DF	*t*-value	*p* value
*Model for aGMV*					
*β*_1_ (days after stroke)	−0.0029	0.0031	21	−0.9516	0.3521
*β*_2_ (age)	−0.0011	0.0021	10	−0.5228	0.6125
*β*_3_ (age: days after stroke)	0.0005	0.0001	21	0.9210	0.3675
*Model for aGMV*_*i*_					
*β*_1_ (days after stroke)	−0.0042	0.0038	21	−1.1104	0.2794
*β*_2_ (age)	−0.0017	0.0023	10	−0.7408	0.4758
*β*_3_ (age: days after stroke)	0.0001	0.0001	21	1.0566	0.3027
*Model for aGMV*_*c*_					
*β*_1_ (days after stroke)	0.0077	0.0036	21	2.1674	**0.0418** ^**∗**^
*β*_2_ (age)	0.0010	0.0023	10	0.4296	0.6766
*β*_3_ (age: days after stroke)	−0.0001	<0.0001	21	−1.9484	0.0649
*Model for LI*_*GMV*_					
*β*_1_ (days after stroke)	0.0139	0.0065	21	2.1194	**0.0461** ^**∗**^
*β*_2_ (age)	0.0034	0.0033	10	1.0442	0.3210
*β*_3_ (age: days after stroke)	−0.0002	0.0001	21	−1.9314	0.0670

^*∗*^ *p* < 0.05.

**Table 3:** Statistical results of linear mixed-effects model for correlation of aGMV_*c*_ and LI_GMV_ with *N*_*c*_ by (6).

	Value	Std. error	DF	*t*-value	*p* value
*Model for aGMV*					
*β*_1_ (*N*_*c*_)	−0.00001	0.00006	22	−1.6819	0.1067
*Model for LI*_*GMV*_					
*β*_1_ (*N*_*c*_)	−0.0003	0.00009	22	−2.8440	**0.0094** ^**∗**^

^*∗*^ *p* < 0.05.

**Table 4:** Statistical results of linear mixed-effects model for correlation of FMI with aGMV by (5).

	Value	Std. error	DF	*t*-value	*p* value
*β*_1_ (aGMV)	−27.1449	11.7658	20	−2.3071	**0.0319** ^**∗**^
*β*_2_ (Age)	−0.8732	0.5009	10	−1.7433	0.1119
*β*_3_ (days after stroke)	0.2971	0.1803	20	1.6482	0.1149
*β*_4_ (age: days after stroke)	−0.0029	0.0029	20	−1.0078	0.3256

^*∗*^ *p* < 0.05.

**Table 5:** Statistical results of linear mixed-effects model for lesion volume by (4).

	Value	Std. error	DF	*t*-value	*p* value
*β*_1_ (days after stroke)	−36.3201	14.6339	21	−2.4819	**0.0216** ^**∗**^
*β*_2_ (age)	−5.5058	12.6501	10	−0.4352	0.6726
*β*_3_ (age: days after stroke)	0.6304	0.2366	21	2.6645	**0.0145** ^**∗**^

^*∗*^ *p* < 0.05.
