# Supplementary material for: Functional Activation-Informed Structural Changes during Stroke Recovery: A Longitudinal MRI Study
Source: Biomed Res Int. 2017 Oct 24;2017:4345205. doi: 10.1155/2017/4345205 (PMC5674725; doi:10.1155/2017/4345205)
Supplement: Supplementary file 1 — Table S1: Subject-wise values for aGMV, aGMVi and aGMVc, LIgmv, N, Ni, Nc, and LIN. Figure S1: The group-level activation map of healthy controls moving their right hands (A); The group-level activation map of stroke patients moving unaffected hands at P1 (B), P2 (C), P3 (D), and P4 (E). The threshold for activation was p < 0.001, cluster size > 13 voxels. Table S2: Statistical Results of Linear Mixed-Effects Model for dynamic change of FMI over time. [file 4345205.f1.pdf]

Supplementary Materials

Table S1. Subject-wise values for *aGMV*, $aGMV_i$ and $aGMV_c$, LI_{gmv}, *N*, N_i, N_c, and LI_N

Subject No.	Time point	*aGMV*	$aGMV_i$	$aGMV_c$	LI_{gmv}	*N*	N_i	N_c	LI_N
1	1	0.31067	0.273321	0.345733	0.116972	914	475	439	-0.03939
	2	0.286923	0.275465	0.307305	0.054636	928	666	262	-0.43534
	3	N/A	N/A	N/A	N/A	N/A	N/A	N/A	N/A
	4	N/A	N/A	N/A	N/A	N/A	N/A	N/A	N/A
2	1	0.368311	0.340946	0.409951	0.091897	1072	767	305	-0.43097
	2	0.309781	0.218848	0.395549	0.2876	907	451	456	0.005513
	3	N/A	N/A	N/A	N/A	N/A	N/A	N/A	N/A
	4	N/A	N/A	N/A	N/A	N/A	N/A	N/A	N/A
3	1	0.284929	0.270376	0.310887	0.069694	1020	567	453	-0.11176
	2	0.354765	0.457781	0.282212	-0.23726	1204	495	709	0.177741
	3	N/A	N/A	N/A	N/A	N/A	N/A	N/A	N/A
	4	N/A	N/A	N/A	N/A	N/A	N/A	N/A	N/A
4	1	0.314618	0.23314	0.3856	0.246405	1152	524	628	0.090278
	2	N/A	N/A	N/A	N/A	N/A	N/A	N/A	N/A
	3	N/A	N/A	N/A	N/A	N/A	N/A	N/A	N/A
	4	N/A	N/A	N/A	N/A	N/A	N/A	N/A	N/A
5	1	0.443945	0.38628	0.512367	0.140307	1072	584	488	-0.08955
	2	N/A	N/A	N/A	N/A	N/A	N/A	N/A	N/A
	3	0.374363	0.360334	0.456782	0.118035	1115	990	125	-0.77578
	4	0.439992	0.474776	0.42487	-0.05547	1062	327	735	0.384181
6	1	0.255153	0.23922	0.317306	0.14031	882	706	176	-0.60091
	2	0.326591	0.288759	0.481291	0.250025	1213	970	243	-0.59934
	3	0.334255	0.260934	0.440542	0.256043	945	575	370	-0.21693
	4	0.282113	0.256857	0.394526	0.211349	913	743	170	-0.6276
7	1	0.406528	0.379546	0.426963	0.058792	1131	417	714	0.262599
	2	0.515092	0.487551	0.570171	0.078112	979	676	303	-0.381
	3	0.400028	0.353699	0.449741	0.119538	1231	623	608	-0.01219
	4	0.409441	0.31718	0.605127	0.312203	966	653	313	-0.35197
8	1	0.28323	0.280015	0.282402	0.004244	1104	792	312	-0.43478
	2	0.331834	0.328376	0.343742	0.022862	1217	660	557	-0.08463
	3	0.286483	0.220378	0.371675	0.255546	999	603	396	-0.20721
	4	0.260932	0.209394	0.36838	0.275169	1133	818	315	-0.44395
9	1	0.340598	0.310249	0.432552	0.164651	963	704	259	-0.4621
	2	0.271305	0.2593	0.35986	0.162413	814	725	89	-0.78133
	3	0.366202	0.301868	0.495547	0.242884	983	649	334	-0.32045
	4	N/A	N/A	N/A	N/A	N/A	N/A	N/A	N/A
10	1	0.28518	0.261404	0.309301	0.083925	946	441	505	0.067653
	2	0.366976	0.329943	0.420364	0.120512	962	568	394	-0.18087
	3	N/A	N/A	N/A	N/A	N/A	N/A	N/A	N/A
	4	N/A	N/A	N/A	N/A	N/A	N/A	N/A	N/A
11	1	0.390734	0.38955	0.39042	0.001116	1149	995	154	-0.73194
	2	0.376578	0.348033	0.417844	0.091152	1025	656	369	-0.28
	3	0.408622	0.402815	0.425347	0.027207	1035	775	260	-0.49758
	4	0.374606	0.336258	0.554005	0.244587	906	747	159	-0.64901
12	1	0.335539	0.359307	0.324886	-0.05031	1204	325	879	0.460133
	2	0.297241	0.270979	0.389	0.178825	1138	885	253	-0.55536
	3	0.296044	0.253874	0.429855	0.257384	1138	861	277	-0.51318
	4	0.304847	0.292627	0.391697	0.144771	1172	1021	151	-0.74232

N/A means the values are not available due to missing fMRI data at corresponding time points.

Results on the first three time points

Analysis was performed for the data of the first three time points as well with the similar analysis method used for the data of all four time points. The dynamic changes of *aGMV*, $aGMV_{c,}$ $aGMV_i$, and LI_{gmv} through three time points as well as their longitudinal relationships with FMI were examined by the linear mixed-effects model. Similar results were found for $aGMV_c$ and LI_{gmv}, that is, their values increased significantly through the first three time points ($aGMV_c$: t = 2.3678, p = 0.0308; LI_{gmv}: t = 2.3990, p = 0.0290). For *aGMV* or $aGMV_i$, no significant dynamic changes were found through the first three time points, which was also similar with the results obtained from the data of all four time points. With respect to the longitudinal relationship of *aGMV*, $aGMV_{c,}$ $aGMV_i$, and LI_{gmv} with FMI, no significant relationship was identified though the relationship between *aGMV* and FMI was close to significance (p = 0.0936).

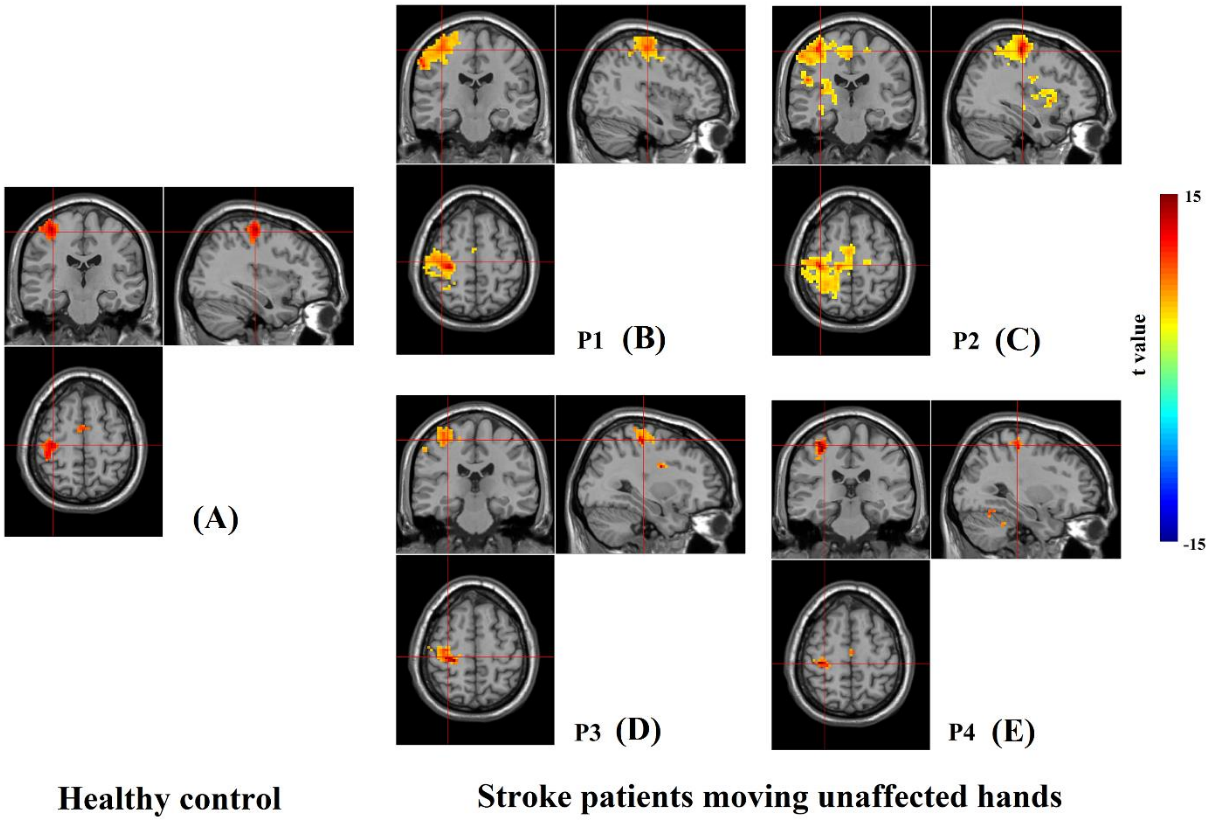

Fig. S1. The group-level activation map of healthy controls moving their right hands (A); The group-level activation map of stroke patients moving unaffected hands at P1 (B), P2 (C), P3 (D), and P4 (E). The threshold for activation was p<0.001, cluster size > 13 voxels.

Dynamic change of FMI over time

The dynamic change of FMI over time was calculated using linear-mixed model, which is as follow:

$$F_{ij} = \mu + b_i + T_{ij}\beta_1 + \varepsilon_{ij}, \; i = 1, 2, 3, \ldots, K; \; j = 1, 2, 3, 4,$$

where F_{ij} represents either the FMI of the *i*th subject from the *j*th scan (up to four scans in this study), μ is the fixed effect term for all patients, b_i is the random effect term for each patient, T_{ij} represents the days post stroke and β_1 is its scalar (the common slope), ε_{ij} represents the residual error of the model, and *K* is the number of patients.

The statistical results are listed in Table S2. The results showed that the FMI increased significantly across four time points ($p < 0.0001$).

Table S2. Statistical Results of Linear Mixed-Effects Model for dynamic change of FMI over time

	Value	Std. Error	DF	*t*-value	*p*-value
β_1	0.1207	0.0172	22	7.0337	<0.0001
